# Changes in Medical Schools

**Published:** 1860-05

**Authors:** 


CHANGES IN MEDICAL SCHOOLS.—New York Medical College: Dr. Horace Green has resigned the presidency of the Faculty of the New York Medical College; and the chairs of Physiology and of Obstetrics and Diseases of Women and Children, in the same institution, have been vacated by the retirement of Dr. Austin Flint, Jr., and Dr. E. R. Peaslee.

Dr. W. A. Hammond, U.S.A., has been appointed Professor of Anatomy and Physiology in the University of Maryland, the chair having been resigned by Professor Roby. On account of the death of Professor Frick, the chair of Materia Medica and Therapeutics, in the same college, has been rendered vacant, but has been filled by the appointment of Dr. Edward Warren, of Edenton, North Carolina.

In the New Orleans School of Medicine, a number of changes have been made. Professor Austin Flint, Jr., has been appointed to the chair of Physiology and Microscopic Anatomy, its former incumbent, Dr. Peniston, having been transferred to that of Anatomy. The latter chair was vacated by Professor Beard, who now becomes Professor of Surgery and Surgical Pathology. Another chair, that of Clinical and Operative Surgery, has been established, to which Professor Choppin has been appointed.

